# From innovation network to public health benefit: evolutionary characteristics and optimization of the biomedical industry innovation network in the Beijing-Tianjin-Hebei region

**DOI:** 10.3389/fpubh.2026.1838874

**Published:** 2026-07-02

**Authors:** Xinfei Xie, Mingmei Xiang, Qingli Tan

**Affiliations:** School of Pharmaceutical Business, Guangdong Pharmaceutical University, Guangzhou, China

**Keywords:** Beijing-Tianjin-Hebei region, biomedical industry, innovation network evolution, obstacle factors, public health translation, regional health equity, social network analysis (SNA), Temporal Exponential Random Graph Model (TERGM)

## Abstract

**Introduction:**

As a core component of strategic emerging industries, the biomedical industry serves as a key indicator of regional scientific and technological innovation capacity and industrial competitiveness. The Beijing-Tianjin-Hebei (BTH) region possesses inherent advantages for development, including abundant research resources, a solid industrial foundation, and vast market potential. However, it also faces structural challenges such as insufficient collaboration within innovation networks and resource misallocation.

**Methods:**

Based on multi-source data, including patent data and enterprise panel data from 2013 to 2023, this paper systematically analyzes the evolutionary characteristics of the BTH biomedical industry innovation network and identifies obstacles to its high-quality development. Methods such as social network analysis (SNA), the Temporal Exponential Random Graph Model (TERGM), and an obstacle degree model are employed.

**Results:**

The study finds that the regional innovation network has evolved from a “dual-core monopoly” (2013–2018) to a “Beijing-Tianjin-Xiong'an triangle structure” emerging by 2023 forming a multi-level collaborative pattern. However, the main obstacles lie in the low efficiency of R&D personnel, insufficient conversion of patent revenue, and inadequate cross-regional policy coordination, with disconnections existing in the “R&D-conversion-industrialization” chain.

**Discussion:**

To address these issues, this paper proposes strengthening the precise division of labor system: “Beijing for R&D, Tianjin for conversion, and Hebei for manufacturing” to improve full-chain innovation services to bridge conversion gaps, optimizing resource allocation to resolve mismatches, and implementing differentiated policies. From a public health perspective, these measures are designed to shorten the R&D-to-registration cycle, lower the cost of essential medicines, and reduce interregional health disparities. By accelerating the translation of biomedical innovations into accessible diagnostics, vaccines, and therapeutics, the optimized innovation network can directly enhance regional health security and epidemic response capacity. This aims to promote high-quality and coordinated regional industrial development, ultimately con tributing to improved public health outcomes by accelerating the translation of R&D into accessible diagnostics, vaccines, and therapeutics, and strengthening regional health security.

## 1 Introduction

As a core component of strategic emerging industries, the biomedical industry serves as a key indicator for measuring regional scientific and technological innovation capabilities and industrial competitiveness. The Outline of the 15th Five-Year Plan explicitly calls for accelerating the development of strategic emerging industries such as the biomedical industry, incorporating them into the list of key emerging pillar industries to be prioritized by the state. In 2026, the Government Work Report first positioned the development of the biopharmaceutical industry as a pillar industry, propelling it into a new phase of historic advancement. The biopharmaceutical industry is not only a critical pillar and key driver in building a Healthy China but also an essential safeguard for addressing changes in disease patterns and enhancing public health emergency response capabilities (1). Since the turn of the millennium, the biomedical industry has become both a primary driver of high-quality economic development and a guardian of public health. Its regional coordinated development directly affects innovation resource allocation efficiency and overall industrial competitiveness (2).

From a public health perspective, biomedical innovation is not an end in itself but a means to achieve faster diagnostic deployment, more accessible vaccines, and stronger epidemic response. As emphasized by Wang et al. (3), biotechnology enables rapid pathogen identification via gene sequencing and early disease detection through molecular diagnostics: tools essential for public health surveillance. Critically, the translation of biomedical R&D into accessible diagnostics, vaccines, and therapeutics depends on the efficiency of regional innovation networks. Efficient networks shorten the cycle from laboratory discovery to clinical application, thereby strengthening a region's health security and its capacity to manage both communicable and non-communicable diseases (3). Empirical evidence from the Yangtze River Delta, Pearl River Delta, and BTH regions confirms that well-connected urban networks significantly enhance synergistic security resilience of regional medical resources (4).Thus, understanding the evolutionary dynamics of biomedical innovation networks is not merely an industrial competitiveness issue but a public health imperative.

The Beijing-Tianjin-Hebei (BTH) region, the most economically vibrant core area in northern China, possesses inherent advantages for biomedical development: abundant research resources, a solid industrial foundation, and vast market potential. Beijing is home to 37 state key laboratories, serving as a cradle for original innovation; Tianjin holds unique strengths in R&D and conversion; and Hebei offers ample industrial space and a robust manufacturing base for industrialization projects (5, 6). However, after over a decade of coordinated development, the BTH biomedical industry still confronts structural contradictions. Significant gradient differences in innovation input-output efficiency persist. Although the technology cooperation network is gradually improving, gaps exist in the “R&D-conversion-industrialization” chain, resulting in a lower conversion rate compared to advanced regions like the Yangtze River Delta. Resource allocation suffers from factor misallocation and spatial imbalance: exemplified by “ample funds but a shortage of high-end talent” in Beijing vs. “sufficient personnel but insufficient funding” in Hebei (7–9). These issues significantly constrain integrated regional development, necessitating systematic research on innovation network evolution and obstacle factors.

From a health system perspective, these structural contradictions are not merely economic inefficiencies. They translate directly into delayed patient access to novel therapies, higher out-of-pocket costs in peripheral cities, and weakened surge capacity for vaccine or therapeutic development during health emergencies (3). As demonstrated during the rapid development of COVID-19 vaccines, efficient innovation networks can compress the R&D-to-deployment timeline from years to months (10). Conversely, fragmented networks with sparse cross-regional linkages risk exacerbating regional health inequities (11, 12). For example, Cai et al. (11) show that fragmented collaboration networks in China's ready-to-cook foods industry: characterized by low network density, which severely limit knowledge diffusion and slow the translation of breakthroughs into applications. Similarly, Wang et al. (13) demonstrate, through analysis of China's new energy vehicle policy networks, that policy coordination significantly influences market outcomes, with network indicators exhibiting strong positive correlations with performance metrics (ρ = 0.800–0.850, *p* < 0.01). Translating these insights to the biomedical domain, we argue that the efficiency of the BTH biomedical innovation network directly affects how quickly R&D outputs are converted into public health goods.

Theoretically, most existing research focuses on a single region or dimension, lacking a coupled analysis of innovation network evolution patterns and input-output efficiency in the BTH region. There is a particular dearth of in-depth analysis on factor flow mechanisms and obstacles under the “core-periphery” structure. By integrating social network analysis with empirical quantitative methods (TERGM, obstacle degree model), this study reveals the node evolution, structural optimization, and driving mechanisms of the BTH biomedical innovation network. Practically, as the BTH coordinated development strategy enters its second decade, the biomedical field still faces unclear functional positioning, impeded factor flow, and low conversion efficiency (5). Utilizing multi-source data from 2013–2023 (patent data, enterprise panel data), this study systematically identifies core obstacles to high-quality development and constructs a regional division-of-labor system characterized by “functional complementarity and staggered coordination.”

**Figure 1:**
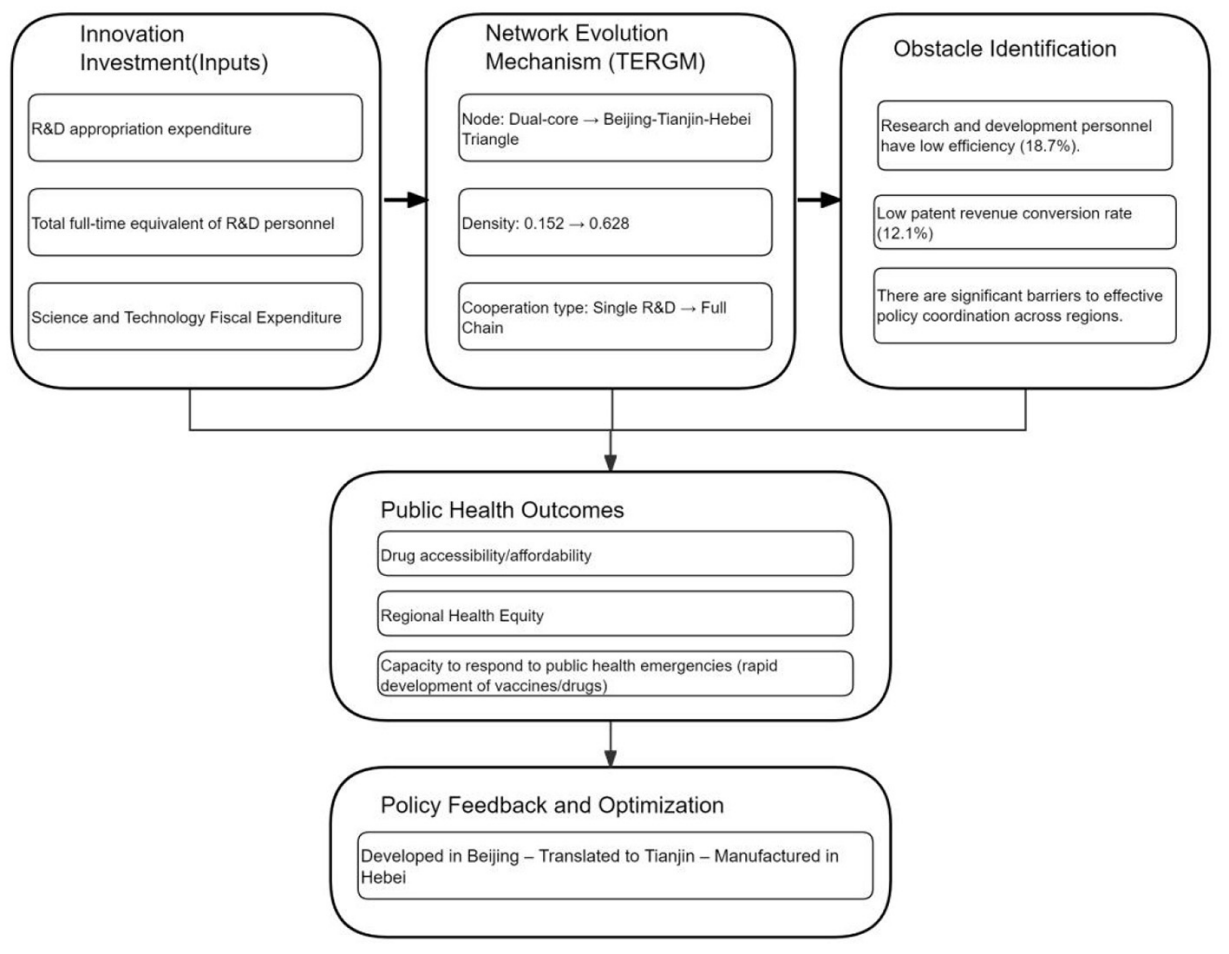
Theoretical framework: biomedical innovation network and public health in the Beijing-Tianjin-Hebei region.

To operationalize the public health implications of our theoretical framework (Figure 1), we propose a causal pathway: Innovation inputs (R&D funding, personnel, fiscal support) determine the quantity and quality of biomedical research outputs. Network evolution (TERGM-identified reciprocity, GDP sender effects, and edge density) shapes how efficiently these outputs diffuse across the BTH region. When networks exhibit sparse, core-periphery structures, peripheral cities face systematic delays in accessing cutting-edge innovations: a phenomenon we term “innovation access inequality.” This inequality translates into public health disparities: delayed patient access to novel therapies, higher out-of-pocket costs in less-connected regions, and weaker surge capacity for vaccine or therapeutic development during health emergencies (e.g., pandemics). Conversely, optimizing the network toward a “Beijing R&D, Tianjin conversion, Hebei manufacturing” division of labor can shorten R&D-to-registration cycles, reduce production costs through manufacturing scale, and enable faster, more equitable distribution of life-saving interventions.

Concretely, the identified obstacles: such as low R&D personnel efficiency and weak patent conversion, are not mere economic inefficiencies; they represent barriers that delay patient access to novel therapies and weaken the region's surge capacity for vaccine development during pandemics.

The core objectives of this study encompass three dimensions: First, to analyze the spatial-temporal characteristics and efficiency disparities of innovation inputs and outputs within the BTH biomedical industry; second, to elucidate the evolution patterns of regional biomedical technology collaboration networks from 2013 to 2023, including their hierarchical structure of key nodes, network architecture, and collaborative models; third, to identify critical factors hindering high-quality development and propose targeted regional coordination mechanisms and optimization strategies. Through empirical analysis of these barriers, we pinpoint the most urgent network bottlenecks requiring policy intervention: low R&D personnel efficiency (18.7%) and insufficient patent commercialization rates (12.1%). Ultimately, by identifying these key obstacles and proposing solutions to bridge the gap between “R&D, translation, and industrialization,” this study aims to accelerate the transformation of biomedical innovations into superior public health outcomes by accelerating the adoption of diagnostic tools, enhancing vaccine accessibility, reducing disease burdens, improving treatment affordability, and strengthening regional health security infrastructure.

## 2 Literature review

### 2.1 Connotation and evaluation of high-quality development in the biomedical industry

The core essence of high-quality development in the biomedical industry lies in achieving the organic unity of innovation-driven growth, efficiency improvement, and optimized benefits. Its connotation encompasses multiple aspects, including industrial scale expansion, innovation capacity enhancement, conversion efficiency improvement, and sustainable development (2, 8). Some studies suggest that the biomedical industry is technology-intensive, with high inputs and risks, requiring synergistic action across the entire “R&D-conversion-industrialization” chain for high-quality development. Core evaluation indicators should include four dimensions: industrial scale, industrial efficiency, industrial innovation, and industrial benefits (14–16). Wan et al. (14), using empirical data from the Yangtze River Delta, constructed an evaluation system encompassing indicators such as total assets, R&D investment intensity, patent applications, and operating profit margin, revealing spatiotemporal differentiation in regional industrial development. Dang et al. (15), from an industrial cluster perspective, incorporated R&D personnel efficiency and technology conversion efficiency into the innovation efficiency evaluation framework, demonstrating the crucial value of factor allocation optimization for high-quality development. Liu et al. (17), further built a relatively mature evaluation system based on existing research. However, research on differentiated evaluation of the BTH region's gradient characteristics remains insufficient, failing to fully reflect the actual industrial development situation under the “core-periphery” structure.

### 2.2 Evolutionary characteristics and driving mechanisms of regional innovation networks

Regional innovation networks are the core carriers of industrial coordinated development, and their evolutionary characteristics and driving mechanisms are hotspots in academic research. The core components of an innovation network include nodes, cooperation links, and network structure (17, 18). Existing research shows that regional innovation network evolution is a dynamic process of node growth, structural optimization, and deepening division of labor, with multi-faceted driving mechanisms: geographical proximity plays a key role in the initial formation of the network, but its influence weakens with the development of transportation and communication technologies (19). Technological similarity and complementarity can determine the depth of cooperation; high similarity lays the foundation for synergy, while low similarity fosters differentiated division of labor (20). Policy synergy, as an institutional guarantee, can effectively break down barriers to factor flow and promote the optimal allocation of innovation resources. Social proximity becomes a core bond in the mature stage of the network, enhancing cooperation stability (21, 22).

In research on biomedical industry innovation networks, Liu et al. (17), using patent data from the Yangtze River Delta, revealed the evolutionary pattern of technology cooperation networks from “core agglomeration” to “region-wide linkage,” confirming the dual driving role of policy synergy and market demand. Wang et al. (18) focused on non-local innovation networks, finding that cross-regional cooperation can enhance innovation quality and that intermediary organizations play a key role in technology transfer. In international studies, Wal (19) used the German biotechnology industry as an example to verify the influence of geographical proximity and triadic closure mechanisms on innovation network evolution. Ge et al. (20). Empirical analysis demonstrates that the network structure of patent-intensive industrial clusters is shaped by both technological compatibility and policy support. While these findings provide a theoretical foundation for this study, the exploration of micro-level mechanisms and node transition pathways in network evolution under the Beijing-Tianjin-Hebei region's “dual-core, two-wings” spatial configuration remains insufficient. This gap constitutes the core focus of our research.

### 2.3 Research on the coordinated development of the biomedical industry in the BTH region

The BTH region is a key urban agglomeration in China, and the coordinated development of its biomedical industry has consistently attracted academic attention. Current research mainly focuses on three dimensions. The first is industrial competitiveness evaluation. Zhuang and Tan (8) constructed an evaluation system based on industrial foundation, innovation capacity, and market environment, finding that the competitiveness of the BTH biomedical industry exhibits a gradient characteristic of “Beijing leading, Tianjin intermediate, Hebei lagging.” Zhang et al. (7), through an analysis of technological innovation strength, demonstrated Beijing's absolute advantages in core technology breakthroughs and patent reserves, while Hebei shows significant industrial growth potential. The second dimension is innovation network structure analysis. In studying the BTH biomedical innovation network, Lv (23) based on industry-university-research cooperation data, pointed out that the network is evolving from a “duopoly” toward “multi-point dispersion,” although overall network density remains relatively low. Further observation from the perspective of patent cooperation networks by Xing and Zhang (24) reveals that while innovation linkages among BTH cities are gradually strengthening, significant internal regional differences persist (42). Research by He and Li (9) confirms this trend, particularly noting that Hebei cities are mostly positioned at the periphery of the innovation network, lacking sufficient core radiative capacity. Regarding obstacles and countermeasures for coordinated development, drawing on the experience of Liaoning's innovation ecosystem construction, Zhao et al. (16) identified insufficient R&D funding, imbalanced talent structure, and lack of conversion platforms as main factors restricting regional industrial development. In their ten-year assessment of BTH coordinated development, Tian and Li (5) further emphasized that administrative barriers and impeded factor flow remain key issues hindering the integrated development of the biomedical industry.

### 2.4 Advances in innovation network analysis: methodological and theoretical insights from patent data

Recent scholarship has substantially advanced the quantitative analysis of innovation networks through patent data, offering valuable methodological and theoretical tools that inform this study. Wang et al. (3) employ the Temporal Exponential Random Graph Model (TERGM) to investigate the evolution of China's biotechnology cooperation networks from 2004 to 2023. Their micromechanisms framework: encompassing agency, opportunity, and inertia, which reveals that network evolution is driven by both endogenous factors (transitivity and convergence) and exogenous factors (geographic homogeneity). Importantly, they demonstrate that policy interventions must account for temporal dependence and lag effects, as cooperative relationships exhibit significant path dependency. This temporal perspective is directly applicable to our analysis of the BTH biomedical network, where we also employ TERGM to capture dynamic evolution patterns.

Cai et al. (11) construct an integrated “space-time-technology-network” analytical framework to examine China's ready-to-cook foods patent ecosystem. Their finding that the collaboration network remains notably fragmented (network density as low as 0.0005) despite explosive patent growth highlights a critical tension: rapid technological output does not necessarily correspond to robust collaborative structures. This insight resonates with our observation of the BTH biomedical network, where increasing patent volumes coexist with persistent “R&D-conversion-industrialization” disconnects. Moreover, their identification of three primary knowledge flow channels: intra-corporate group collaborations, industry-university-research partnerships, and regional industrial clusters. These provide a typology that informs our analysis of cooperation patterns in the biomedical sector.

Wang et al. (13) combine social network analysis with Granger causality tests to evaluate China's new energy vehicle policy networks. Their finding that network metrics (density, average path length, transitivity, clustering coefficient, and largest component size) exhibit strong positive correlations with market performance (ρ = 0.800–0.850, *p* < 0.01) and that largest component size has an immediate causal effect (*F* = 4.152, *p* < 0.05) offers a rigorous methodological template for linking network structure to outcomes. Translating this to the biomedical domain, we hypothesize that similar causal pathways exist between policy coordination and public health outcomes: a hypothesis we partially test through our obstacle degree model and TERGM analysis.

Finally, Cai et al. (25) employ BERTopic modeling and social network analysis to examine China's nuclear fusion engineering innovation system, proposing the concept of “organized fragmentation,” a governance mode where complex big science projects are decomposed into vertically integrated but horizontally segregated sub-clusters under strategic state direction. Their finding that the Chinese system exhibits a “dual-core drive” structure with high modularity (0.858) and that research institutes act as “super-hubs” controlling information flow (with betweenness centrality 0.0228 for the top institute) offers a theoretical lens for understanding the BTH biomedical network's core-periphery structure. The concept of “organized fragmentation” helps explain why a seemingly sparse network can still achieve significant technological output: modular division of labor, rather than dense connectivity, may be the efficient organizational form for mission-oriented innovation in resource-constrained contexts.

### 2.5 Research on obstacle factors and regional synergy mechanisms in the biomedical industry

The high-quality development of the biomedical industry is constrained by multiple factors. Existing research generally agrees that insufficient R&D investment, imbalanced talent structure, low technology conversion efficiency, and inadequate policy synergy are the most core obstacles (9). In a study of Liaoning's biomedical industry, Zhao et al. (16) found that issues like an imperfect innovation ecosystem, missing pilot-scale test links, and insufficient clinical trial resources severely impact industrial competitiveness. He and Li (9) pointed out structural obstacles in the BTH biomedical innovation network, such as the “disconnect between R&D and industry” and “loose regional cooperation.” At the factor allocation level, the mismatch of capital and talent is particularly prominent. Beijing faces a situation of “surplus funds but a relative shortage of high-end talent,” while Hebei is caught in a dilemma of “sufficient personnel but insufficient funds.” This factor mismatch significantly reduces the overall innovation efficiency of the region (8, 21).

In response to these obstacles, academia has proposed various synergy mechanisms. Regarding industry-university-research collaboration, based on the Yangtze River Delta experience, Wan et al. (26) confirmed that patent cooperation and project funding can effectively promote the integration of innovation resources and enhance industrial integration (43, 44). In terms of policy synergy, Tian and Li (5) emphasized the need to establish cross-regional policy coordination mechanisms to break down administrative barriers and promote the free flow of innovation factors. Concerning platform construction, Liu and Zheng (27), through a study of policy evolution in the Chengdu-Chongqing region, pointed out that the construction of carriers like technology trading platforms and pilot-scale test bases is key to bridging the “R&D-conversion” gap. However, existing research lacks sufficient specificity for the BTH region and fails to fully integrate its gradient division of labor characteristics: “Beijing R&D, Tianjin conversion, Hebei manufacturing” to propose synergistic mechanisms that are both systematic and actionable.

Overall, existing research has preliminarily revealed the development status, network characteristics, and some obstacle factors of the BTH biomedical industry. However, significant research gaps remain. First, there is a lack of coupled analysis between innovation input-output efficiency and innovation network evolution, making it difficult to explain the core contradiction of “converging input scale but diverging efficiency.” Second, obstacle factor identification is often concentrated on a single dimension, lacking systematic deconstruction from a full industrial chain perspective. Third, countermeasures for coordinated development are mostly macro-oriented, with insufficient design of differentiated mechanisms based on network evolution patterns and regional gradient characteristics. Consequently, this study, grounded in multi-source data and multiple methods, systematically analyzes the evolutionary characteristics and obstacle factors of the BTH biomedical innovation network, aiming to provide more precise theoretical support and practical pathways for the high-quality coordinated development of the regional industry.

### 2.6 From industrial innovation to public health benefit: a theoretical bridge

The translation of biomedical research achievements into health benefits for populations largely depends on the operational efficiency of regional innovation networks (12). Previous studies have revealed that low patent commercialization efficiency directly hinders timely access to novel diagnostic methods and therapeutic approaches for patients (28, 29, 45). Furthermore, inadequate allocation of R&D personnel and underdeveloped cross-regional collaboration mechanisms drive up drug production costs, which are ultimately passed on to patients or healthcare systems (30, 31).

From a policy coordination perspective, the hierarchical healthcare system and vaccine incentive mechanisms offer valuable insights for biomedical innovation networks (30, 32). Just as tiered healthcare delivery enhances resource allocation efficiency, a clear division of labor among “research and development-transformation-production” can reduce redundant investments and accelerate the supply of essential medicines in underdeveloped regions (47).

It is noteworthy that the spatial structure of innovation networks directly determines health equity. Marginal cities in the BTH region (such as several prefecture-level cities in Hebei Province) may experience systemic delays in accessing cutting-edge biomedical innovations from Beijing and Tianjin (12). This “inequality in innovation access” represents an underexplored factor contributing to regional health disparities. Recent assessments of regional medical resource synergy further suggest that city network structures directly influence health system resilience, particularly during public health emergencies (4).

## 3 Data sources and methodology

### 3.1 Research methods

#### 3.1.1 Construction of the indicator system

Drawing on the secondary indicator evaluation framework for high-quality development in the biomedical industry constructed by Wan et al. (14) and considering the core characteristics of innovation input-output in the BTH industry, a comprehensive evaluation index system is constructed from four dimensions: industrial scale, industrial efficiency, industrial innovation, and industrial benefits. The indicator attributes, units, and connotations are clearly defined, as shown in Table 1.

**Table 1:** Construction of the Indicator System.

Primary indicator	Secondary indicator	Code	Indicator explanation	Unit	Attribute
Industrial scale A	Number of pharmaceutical enterprises	a1	Total number of biomedical industry enterprises in BTH	Count	+
	Total assets of pharmaceutical Mfg.	a2	Total assets of biomedical enterprises	10,000 yuan	+
	Operating revenue of pharmaceutical Mfg.	a3	Total main business revenue of biomedical enterprises	10,000 yuan	+
Industrial efficiency B	R&D expenditure intensity	b1	R&D Expenditure/Total Assets	%	+
	R&D personnel efficiency	b2	Patent Applications/R&D Personnel Full-time Equivalent	piece/Person-year	+
	Technology conversion efficiency	b3	Technology Market Turnover/R&D Expenditure	Ratio	+
Industrial innovation D	R&D expenditure	d1	Total internal + external R&D expenditure	10,000 yuan	+
	R&D personnel full-time equivalent	d2	actual annual equivalent working hours of R&D personnel	Person-year	+
	Number of patent applications	d3	Total annual invention + utility model patent applications	Count	+
	Science & technology fiscal expenditure	d4	Government science and technology support funds for the industry	10,000 yuan	+
Industrial benefit C	Operating profit margin	c1	Operating Revenue of Pharmaceutical Mfg./Total Assets	%	+
	Patent revenue conversion rate	c2	Operating Revenue of Pharmaceutical Mfg./Patent Applications	10,000 yuan/piece	+
	Technology transaction contribution rate	c3	Technology Market Turnover/Operating Revenue	%	+

“+” indicates a positive indicator.

This paper uses the moving average method to impute missing data. Monetary indicators are deflated using 2018 as the base period to eliminate price fluctuation effects. The range method is used to standardize the indicators, unifying the data dimensions.

The standardization formula for positive indicators is:


$$\begin{array}{l} r_{ij}=\frac{y_{ij}-\min\left(y_{ij}\right)}{\max\left(y_{ij}\right)-\min\left(y_{ij}\right)} \end{array}$$

Where r_*ij*_ is the standardized value of the *j*-th secondary indicator under the *i*-th primary indicator, y_*ij*_ is the original value of the indicator, and max(y_*ij*_), min(y_*ij*_) are the maximum and minimum original values of that indicator, respectively.

#### 3.1.2 Empirical analysis of innovation network evolution mechanism based on TERGM

To reveal the internal dynamics of the BTH biomedical technology cooperation network evolution, this study draws on the “structure-attribute-dynamics” analysis framework proposed by Liu et al. (17) and constructs a Temporal Exponential Random Graph Model (TERGM). This model systematically tests the impact of network structural effects, node attribute effects, and dynamic dependence effects on the formation and maintenance of cooperative relationships, providing an empirical basis for optimizing the regional innovation network. Compared to traditional static network models (e.g., ERGM), TERGM can incorporate time-dependent terms, effectively capturing the path-dependent characteristics of network evolution, making it suitable for analyzing multi-period panel network data. The basic form of the model is:


$$\begin{array}{l} {\text{net}}_{t}\sim \text{TERGM}\left(\text{edges}+\text{nodecov}\left(\text{X}\right)+\text{mutual}+\text{Autoregression}\right) \end{array}$$

Where *net*_*t*_ represents the technology cooperation network in period *t*. The explanatory variables cover three core effects: network structural effects (edges, mutual, Dgwesp.OTP), node attribute effects [nodecov (X)], and dynamic dependence effects (Autoregression).

Model Specification and Robustness Discussion. To address potential endogeneity concerns, particularly reverse causality where network cooperation might also influence a city's GDP (gdp), our TERGM specification conditions the formation of network ties at time *t* on node attributes (e.g., GDP, fiscal expenditure) measured at time *t*-1. This temporal precedence structure mitigates reverse causality by ensuring that the explanatory variables precede the network ties they are modeling. Furthermore, the model includes a memory effect through an autoregressive term (Autoregression), which accounts for the path dependency of network evolution, i.e., the likelihood of a tie at time *t* is conditional on the network structure at time *t*-1. A first-order lag (lag = 1) was chosen based on the assumption that policy adjustments and economic shifts typically take at least one year to influence R&D collaboration decisions, aligning with the annual resolution of our panel data. A first-order lag was preferred over higher-order lags because preliminary analysis showed that the autocorrelation function of the network's edge count decayed sharply after one year (partial autocorrelation coefficient fell from 0.63 at lag 1 to 0.11 at lag 2), indicating that direct temporal dependence beyond one year is negligible for this dataset. Including the mutual (reciprocity) term also helps control for unobserved dyadic dependencies that could otherwise bias the estimates of exogenous attribute effects.

Referring to the “four-dimensional variable classification method” (17), and combining it with the characteristics of BTH data, the variables are defined as follows (Table 2).

**Table 2:** Variable settings and interpretation of the TERGM model.

Type of variable	Variable name	Measuring method, method of measurement, philosophy of measurement	Theoretical expectation
Network infrastructure	Edge density	The Ratio of the Actual to the Potential Number of Cooperative Relationships in a Network	- (Control network sparsity)
Endogenous structural variable	Mutual	The Trend of the Formation of Two-way Cooperation between Cities	+ (Collaboration Enhanced)
Exogenous attribute variable	Economic endowment (GDP)	Annual GDP of the city (in ten thousand yuan, adjusted for inflation)	+ (Economic foundation support)
Exogenous attribute variable	Policy support (gov_exp)	Financial expenditure on urban science and technology (10,000 yuan)	+ (Policy Guidance and Cooperation)

In the configuration of the TERGM model, node attributes (GDP and gov_exp) are modeled as sender effects (also referred to as “nodecov” effects in the ERGM software package). Specifically, for each directed binary relationship (*i*→*j*), the model incorporates attribute values of the sending city *i*. This modeling approach reflects the following assumption: regardless of recipient attributes, cities with higher economic output or greater fiscal investment in technology are more likely to establish cooperative relationships. We tested alternative model specifications (e.g., recipient effects, absolute difference matching effects) and found that the sender effect model achieved the best fit (with the lowest AIC value). This choice is theoretically justified: within the Beijing-Tianjin-Hebei context, Beijing's substantial economic scale and robust fiscal capacity position it as a primary hub for technology exports, which aligns perfectly with the sender effect explanation.

**Figure 2:**
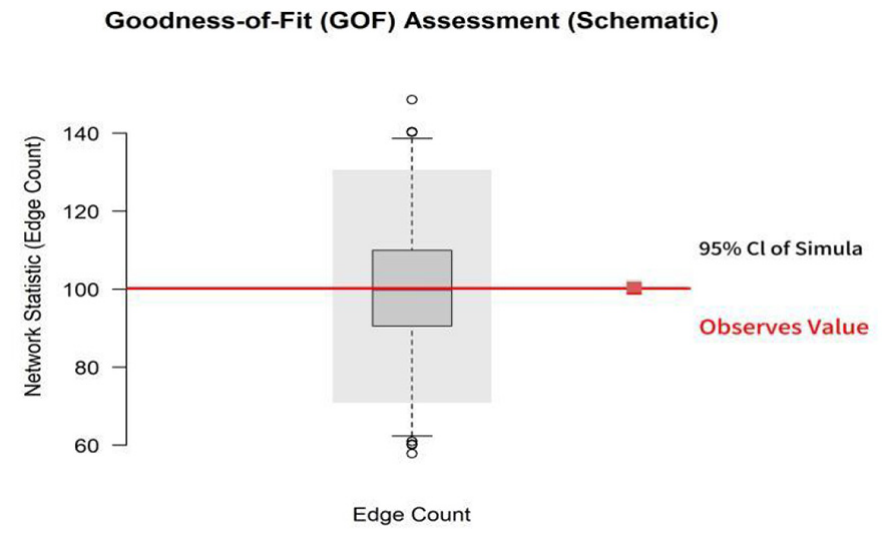
Schematic goodness-of-fit assessment.

Model convergence and small-sample robustness. Given the moderate size of our network (13 nodes across the three time points), we took several precautions to ensure the statistical reliability of the TERGM estimates. First, we used a parametric bootstrap with 1,000 replications to obtain robust standard errors and confidence intervals. Second, to mitigate potential bias due to the small number of nodes, we adopted a first-order autoregressive term (Autoregression), conditioning each time step's network on the previous step's network. Third, we assessed MCMC convergence using the Geweke diagnostic; all parameters showed |Z| < 1.96, indicating successful convergence (see Table 3). Fourth, we conducted a goodness-of-fit (GOF) assessment by simulating 1,000 networks from the fitted model and comparing their edge count distribution to the observed value. The observed edge count fell within the 95% confidence interval of the simulated distribution, confirming that the model is not degenerate and fits the data well (Figure 2). These procedures collectively address the potential estimation concerns associated with network sparsity and small sample size.

**Table 3:** TERGM convergence diagnostics (Geweke Z-scores).

Parameter	Geweke Z–score	Convergence (|Z| < 1.96)
edges	−0.83	Yes
mutual	1.21	Yes
gdp	0.45	Yes
gov_exp	−0.67	Yes

The gray boxplot represents the distribution of edge counts simulated from 1,000 networks generated by the fitted TERGM. The red horizontal line is the observed edge count in the actual network, and the gray shaded area indicates the 95% confidence interval of the simulated distribution. The observed value lies within the confidence interval, indicating no degeneracy and acceptable model fit.

#### 3.1.3 Descriptive statistical analysis

Used to reveal the distribution characteristics of indicator data. Core indicators include skewness and kurtosis.

Skewness formula:


$$\begin{array}{l} SK=\frac{\text{n}}{\left(n-1\right)\left(n-2\right)}{\overset{n}{\underset{i=1}{\sum }}\left(\frac{x_{i}-\bar{x}}{s}\right)}^{4}-\frac{3{\left(n-1\right)}^{2}}{\left(n-2\right)\left(n-2\right)} \end{array}$$

Where *n* is the sample size, $\bar{x}$ is the sample mean, and *s* is the standard deviation. *SK*>0 indicates a right-skewed distribution, *SK*≈0 indicates an approximately symmetric distribution.

Kurtosis formula:


$$\begin{array}{l} K=\frac{n\left(n+1\right)}{\left(n-1\right)\left(n-2\right)\left(n-3\right)}\overset{n}{\underset{i=1}{\sum }}\left(\frac{x_{i}-\bar{x}}{s}\right)-\frac{3{\left(n-1\right)}^{2}}{\left(n-2\right)\left(n-3\right)} \end{array}$$

Where *K*≈3 indicates a distribution close to normal, *K*>3 indicates a leptokurtic distribution (sharp peak), and *K* < 3 indicates a platykurtic distribution (flat peak).

#### 3.1.4 Obstacle degree model

The core purpose of using the obstacle degree model is to diagnose and identify various factors affecting industrial development, thereby accurately pinpointing the most critical and prominent constraints. By quantitatively analyzing, this model can effectively distinguish the impact of different obstacle factors, clarifying the main shortcomings that restrict industrial upgrading and coordinated development. This provides an empirical basis for subsequent targeted policy recommendations and solutions. The calculation formula is as follows:


$$\begin{array}{l} G_{ij}=\frac{\left(1-N_{ij}\right)W_{ij}}{\sum_{i=1}^{4}\sum_{j=1}^{k}\left(1-N_{ij}\right)W_{ij}}\times 100\% \end{array}$$

Where G_*ij*_ is the obstacle degree of the *j-*th secondary indicator under the *i*-th primary indicator, N_*ij*_ is the standardized value of the indicator, and W_*ij*_ is the comprehensive weight of the indicator, determined using a combination of the CRITIC method (for objective weighting) and the Analytic Hierarchy Process (AHP, for subjective weighting). The calculated weights for all secondary indicators are presented in Table 4. All AHP judgment matrices passed the consistency check, with Consistency Ratios (CR) < 0.1, indicating acceptable consistency.

**Table 4:** Comprehensive weights (W_ij_) of evaluation indicators based on AHP-CRITIC method.

Secondary indicator (Code)	AHP weight	CRITIC weight	Comprehensive weight (W_*ij*_)
R&D personnel efficiency (b2)	0.32	0.41	0.365
R&D expenditure (d1)	0.28	0.35	0.315
Patent revenue conversion rate (c2)	0.22	0.18	0.200
Enterprise agglomeration (a1)	0.18	0.06	0.120

The AHP weights were derived from expert surveys, and the Consistency Ratio (CR) for the judgment matrix was 0.052 (< 0.1), confirming the consistency of the subjective evaluations. The CRITIC weights were calculated based on the data's contrast intensity and conflict. The comprehensive weights (W_*ij*_) are the normalized product of the AHP and CRITIC weights.

### 3.2 Data sources and description

This study primarily analyzes patent application data in the biomedical field within the BTH region from 2013 to 2023 to examine innovation trends and R&D activities. To clearly illustrate the phased evolution of the network, three representative time points were selected policy initiation phase, collaboration deepening phase, and high-quality development transition phase (2013, 2018, 2023) for visualization and comparison of key indicators. Data mainly originate from the *Beijing Statistical Yearbook, Tianjin Statistical Yearbook, Hebei Statistical Yearbook* (2013–2023), and the IncoPat global patent database. Additionally, the registered capital data for 53 biomedical enterprises in the Tianjin-Hebei area were supplemented using the enterprise business information database. According to the classification of biomedical industry content in the *Strategic Emerging Industry Classification (2018)* issued by the National Bureau of Statistics, the biomedical industry encompasses the manufacturing of biological pharmaceutical products, manufacturing of chemical drugs and raw materials, manufacturing of modern Chinese medicines and ethnic drugs, manufacturing of key equipment and raw materials for biomedicine, and related biomedical services. This study adopts a definition consistent with the above classification: the biomedical industry is an industry based on life sciences and biotechnology (referencing the “*14th Five-Year” Bioeconomy Development Plan*), integrating theoretical and technical means such as informatics, systems science, and engineering control, engaged in the R&D, production, and sales of products for prevention, treatment, diagnosis, and rehabilitation, involving both the biotechnology industry and the pharmaceutical industry.

## 4 Analysis of the evolutionary characteristics of the BTH biomedical technology cooperation network

Based on policy promotion, the deepening of industrial collaboration, and the transformation of development stages, this study selects 2013, 2018, and 2023 as the analytical nodes: 2013 marks the critical starting point of the Beijing-Tianjin-Hebei coordinated development strategy. Prior to this, the biomedical industries in the three regions faced development barriers, each focused on its own agenda. This node signifies the transition of industrial collaboration from concept to policy layout and serves as the origin of innovation network construction. In 2018, the “Beijing-Tianjin-Hebei Pharmaceutical Industry Association Industrial Park Alliance” was established, coupled with the implementation of earlier policies such as the “off-site supervision” in the Beijing-Cangzhou Bohai New Area Biomedical Industrial Park. This marked the entry of industrial transfer, undertaking, and resource integration into a substantive promotion phase, with the innovation network shifting from preliminary construction to deepening collaboration. In 2023, not only were policies intensified with the introduction of Hebei's “1+3” policy system for biomedicine and the joint release of a work plan for the transformation of scientific and technological achievements by the three regions, but it was also a critical period for post-pandemic industrial recovery and the transition toward high-quality development. At this time, the innovation network had reached a certain scale, while deeper collaborative obstacles became more prominent. These three nodes, spaced approximately five years apart, correspond to the “policy initiation period,” “collaboration deepening period,” and “high-quality development transition period,” respectively. They can clearly present the evolutionary trajectory of the BTH biomedical industry innovation network from non-existence to existence and from loose to tight, accurately capturing the development dynamics and core obstacles brought by factors such as policies, markets, and mechanisms at different stages, thus providing sample support with both continuity and representativeness for the analysis.

Based on core data including patent data and urban cooperation edge lists for the BTH biomedical industry in 2013, 2018, and 2023, the TERGM model was used to analyze the cooperative evolution mechanism of the BTH biomedical industry innovation network. The results are presented in Table 5.

**Table 5:** Key parameter results.

City pair	Patent similarity (Jaccard)	Cooperation characteristics
Beijing-Tianjin	0.6667	Moderately high similarity, good cooperation foundation
Beijing-Hebei	0.5385	Moderate similarity, favorable foundation for R&D-manufacturing division
Tianjin-Hebei	0.5385	Moderate similarity, synergy potential in translation and manufacturing

Jaccard similarity coefficient (based on the IPC classification number set).

**Figure 3:**
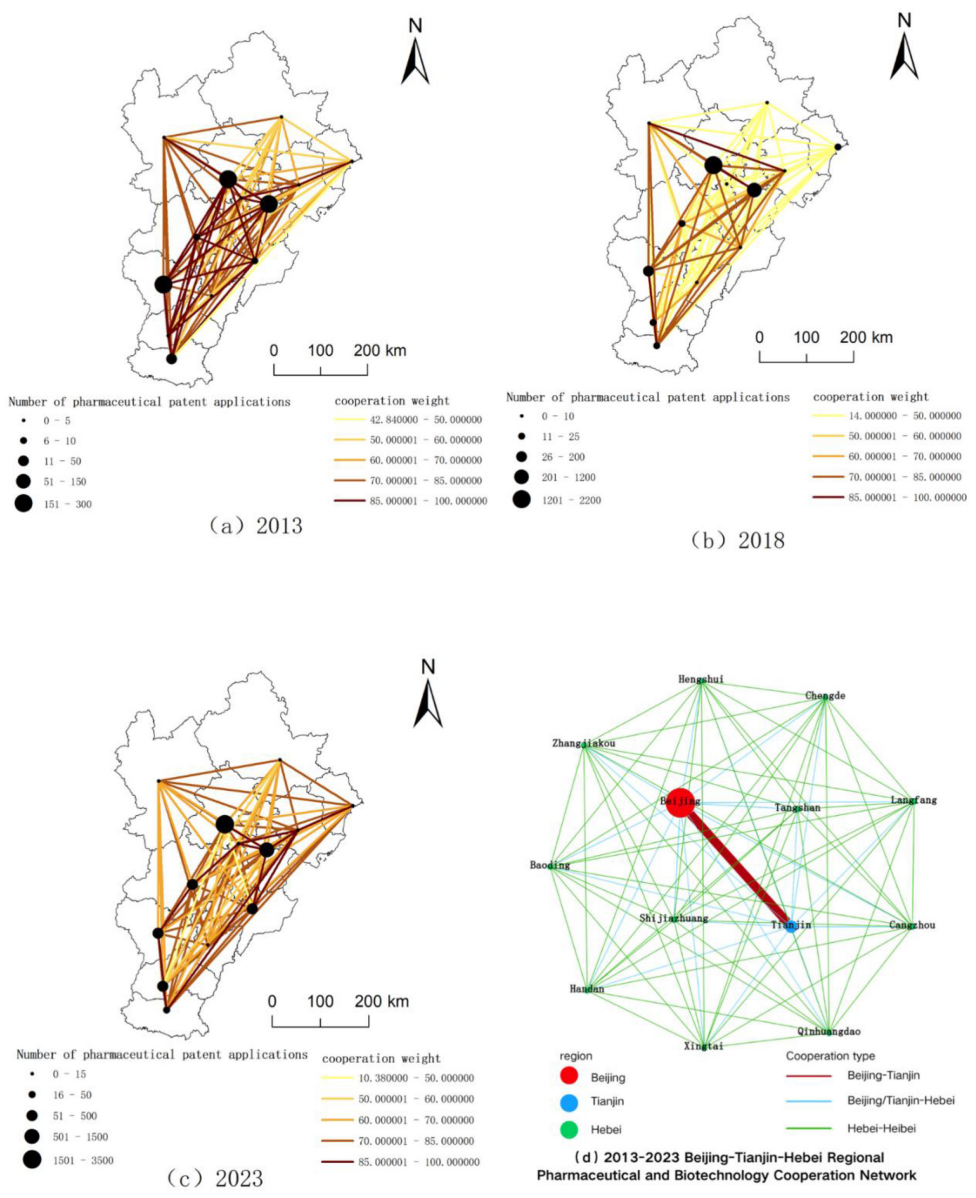
Evolution of the biomedical industry innovation network in the Beijing-Tianjin-Hebei region (2013, 2018, 2023).

Using ArcGIS and Gephi software for visualization, combined with social network analysis and a multi-factor comprehensive weighting model, this study systematically analyzes the evolutionary characteristics and internal mechanisms of the BTH biomedical technology cooperation network from multiple dimensions: core node hierarchy, overall network structure, cooperation patterns and types, and interaction modes. It reveals the dynamic development laws of regional collaborative innovation, providing theoretical support and practical reference for high-quality industrial development (14, 17, 18). The results are detailed in Figure 3.

### 4.1 Evolution of core nodes and hierarchical structure: from duopoly to Beijing-Tianjin-Xiong'an tripod support

The evolution of core nodes exhibits phase characteristics of “core consolidation, hierarchical enrichment, and enhanced radiation,” aligning well with the BTH “dual-core, two wings, multi-node” spatial development layout, constructing a multi-level network system with a clear gradient and complementary functions (24, 33) (Table 6).

**Table 6:** Classification criteria for core node weight levels.

Weight level	Score range	Technology match	Economic association characteristics	Cooperation frequency	Partner positioning
S–level	85–100 points	≥0.7	Close	≥15 times	Core Cooperation Partner
A–level	70–84 points	0.5–0.7	more closely integrated, with clearly defined collaborative platforms	8–14 times	Key Cooperation Partner
B–level	60–69 points	0.3–0.5	General, strong willingness to cooperate	3–7 times	Potential Cooperation Partner
C–level	50–59 points	0.2–0.3	Weak, foundation needs cultivation	1–2 times	Cautious Cooperation Partner
D–level	Below 50 points	< 0.2	Weak	No substantive cooperation	Suspend Cooperation Partner

In 2013, the network exhibited a typical “dual-core monopoly” situation, with only Beijing and Tianjin entering the S-level core nodes. Their technology match in key areas such as pharmaceutical preparations and specific therapeutics reached 0.88. Within Hebei Province, only Shijiazhuang was a B-level node, with all other cities classified as C/D-level. At this stage, leveraging their strong scientific research resources and industrial base, Beijing and Tianjin formed an absolute core position, with network resource allocation displaying prominent polarization characteristics. Most cities in Hebei were at the periphery, and the core's radiative capacity was confined to Beijing and Tianjin (9, 33) (Table 7). The formation of this pattern was closely related to the Matthew effect in regional innovation capacity, with innovation resources highly concentrated in core cities and peripheral cities lacking effective undertaking carriers (22). Network resource allocation exhibited significant polarization characteristics (23, 34).

**Table 7:** Innovation resource allocation and hierarchical characteristics of core nodes.

Core node type	Representative cities	S&T Fiscal Exp. (10 thousand yuan)	R&D Exp. (10 thousand yuan)	Patent apps (Annual Avg.)	Tech strength	Hierarchical positioning	Evolutionary characteristics
Primary Core Node	Beijing	3,802,750.55	361,086.55	754.36	11,955	Core Cooperation Partner	Continuously consolidating R&D leadership
Primary Core Node	Tianjin	1,037,935.73	167,445.69	778.36	3,231	Core Cooperation Partner	Strengthening achievement conversion hub role
Secondary Key Node	Shijiazhuang, Baoding	167,300.45/ 125,475.34	44,734.95/ 33,551.22	178.16/133.62	25/19	Key Cooperation Partner	Taking the Core Radiation, Technology Leap
Tertiary Potential Node	Tangshan, Langfang, etc.	150,570.41/ 100,380.27	40,261.46/ 26,840.97	160.34/106.90	22/15	Potential Cooperation Partner	Differentiated Embedded Cooperative Network
Emerging Core Node	Xiong'an New Area (2023)	–	–	–	–	Core Cooperation Partner	Innovation growth pole in high–end fields

Xiong'an New Area is a national-level new district under the jurisdiction of Hebei Province, established in 2017. It was only identified as an emerging core in the 2023 analysis; however, it was not separately listed as a network node in that analysis.

By 2018, the hierarchical structure of core nodes was preliminarily improved. The S-level core status of Beijing and Tianjin continued to consolidate, while resource control capacity developed toward a multi-point dispersion situation. Hebei Province gained four A-level nodes: Baoding, Shijiazhuang, Langfang, and Cangzhou. Among these, Baoding, leveraging its locational advantage, achieved a technology match of 0.68, becoming Hebei's preferred partner city. B-level nodes expanded to five, and the core's radiative range gradually extended toward central and southern Hebei (6, 21). This change was facilitated by the advancement of the Beijing-Tianjin-Hebei coordinated development strategy, gradually breaking down barriers to the flow of regional innovation factors and steadily enhancing the innovation capacity of peripheral cities, which began actively embedding themselves into the core network (7, 35).

By 2023, Baoding and Xiong'an New Area in Hebei entered the S-level node, forming the “Beijing-Tianjin-Hebei” triangular core pattern. The technology match between Beijing and Xiong'an New Area in high-end fields such as gene therapy and biological vaccines reached 0.85, making them a regional innovation growth pole (8, 35). A-level nodes increased to eight, with cities like Tangshan, Handan, and Zhangjiakou achieving hierarchical upgrading. B-level nodes covered all prefecture-level cities in Hebei. Betweenness centrality exhibited a “three pillars” situation of Beijing, Tianjin, and Hebei Xiong'an New Area, with Langfang and Cangzhou's betweenness centrality both exceeding 0.2. Network resource allocation became increasingly balanced, and the coordinated regulation pattern of hub nodes was formally established (9, 22).

Core nodes achieved a diversified layout, with all Hebei prefecture-level cities entering the potential cooperation partner level, forming a coordinated regulation pattern of hub nodes (36, 37). This evolutionary outcome reflects the reconstruction of the regional innovation pattern driven by policies and also embodies the differentiated development achieved by non-core cities through technological accumulation. Network resource allocation transitioned from concentration to equilibrium, laying the foundation for region-wide synergy (38, 40).

### 4.2 Evolution of overall network structure: from decentralized isolation to region-wide synergy in BTH

The evolution of the BTH biomedical technology cooperation network from 2013 to 2023 reflects a fundamental transition from unidirectional technology transfer to balanced, multi-polar collaboration (5, 17).

In 2013, the network was at an initial agglomeration stage. With five zero-patent cities and a density of only 0.615, the structure exhibited a typical core-periphery pattern where technology flowed unidirectionally from patent-active cities (led by Tianjin) to peripheral recipients (9, 33). The high output concentration (CV = 0.792) and pure technology-transfer mode indicated weak indigenous innovation capacity and limited reciprocal linkages (2, 39) (Table 8).

**Table 8:** Key indicators of overall network structure evolution.

Evolution stage	Year	Positive patent cities	Zero-patent cities	Directed edges	Network density	Total cooperation weight	Avg weighted out-degree	Out-degree CV	In-degree CV
Initial agglomeration	2013	8	5	96	0.615	7,793.63	599.51	0.792	0.098
Diffusion & adjustment	2018	10	3	120	0.769	5,863.72	451.06	0.608	0.406
Comprehensive synergy	2023	12	1	144	0.923	10,550.48	811.58	0.295	0.0596

The network is modeled as a directed graph with 13 fixed nodes (all prefecture-level and above cities in the BTH region). Edge construction follows a “core-periphery” rule: (1) Between any two patent-active cities, cooperation is bidirectional (two directed edges). (2) From any patent-active city to any zero-patent city, only a one-way outgoing edge (technology transfer) is created. (3) No edge is created from a zero-patent city to any other city. This specification realistically captures that zero-patent cities only receive, but do not initiate, technology transfers. Directed density = (actual directed edges) / [N × (N−1)], with *N* = 13.

By 2018, the network entered a diffusion and adjustment phase. The number of patent-active cities increased to ten, and density rose to 0.769, signaling expanded coverage (5, 24). However, total cooperation weight declined, and the weighted in-degree CV jumped to 0.406, revealing a Matthew effect: resources became more concentrated in hubs like Shijiazhuang, while some peripheral cities lagged (7, 22). This stage reflects the growing pains of rapid expansion, where nascent R&D cooperation began to emerge but overall synergy remained fragile (14, 37).

In 2023, the network achieved comprehensive synergy. With density reaching 0.923 (near-full connectivity) and only one zero-patent city left, the structure became highly balanced: both weighted out-degree and in-degree CVs dropped to below 0.3 (8, 17). Tangshan emerged as a new core alongside Beijing, Tianjin, and Shijiazhuang, forming a multi-polar pattern. Cooperation evolved into composite models integrating R&D, patents, and technology transfer, reflecting deep regional integration (5, 26).

This evolutionary trajectory from decentralized hierarchy to dense equality fully demonstrates the successful transformation of the BTH Biomedical Innovation Network from a “central-radiating model” to a “networked collaborative paradigm.” This shift has been driven by coordinated policy efforts, infrastructure development, and increasingly robust social connections (19, 21, 39).

### 4.3 Evolution of cooperation patterns and spatial structure: from Beijing-Tianjin axis-driven extension to region-wide cluster development

The evolution of cooperation relationships revolves around the three cores of “spatial compression, domain expansion, and intensity enhancement.” The spatial pattern deeply aligns with the spatiotemporal distance optimization effect of the “Rail-on BTH,” achieving a strategic transformation from a “Beijing-Tianjin axis” to “dual-axis driving, region-wide linkage” (5, 24). Its essence is the inevitable result of the deepening regional industrial division of labor and the upgrading of innovation demands.

In 2013, cooperation exhibited a pattern of “single-point agglomeration in Beijing-Tianjin, blank periphery.” High-strength cooperation existed only in the Beijing-Tianjin pair, medium-strength cooperation was absent, and low-strength cooperation accounted for 100%. The Beijing-Shijiazhuang cooperation weight was low, internal Hebei and cross-regional cooperation were weak, and technology fields were confined to traditional medicine categories (9, 33). The cooperation radius was limited to within 100 km, with a spatial attenuation coefficient of 0.76, making distance a major factor restricting cross-regional cooperation. Regional industrial homogenization competition was evident, lacking effective support for high-end innovation cooperation (34, 37) (Table 9).

**Table 9:** Core characteristics of spatial structure evolution.

Evolution stage	Year	Cooperation radius (km)	Spatial attenuation coefficient	Core spatial structure	Main cluster plates	Characteristics of high-strength cooperation links
Axial Driving	2013	≤ 100	0.76	Beijing-Tianjin single axis	Beijing-Tianjin core cluster	High-strength cooperation only between Beijing and Tianjin
Dual-Axis Expansion	2018	≤ 200	0.52	B-T axis + B-B-S secondary axis	B-T core + embryonic Beijing-adjacent collaboration	Linkage between B-T and key central-southern Hebei cities
Region-wide Cluster	2023	≤ 300	0.31	Region-wide coverage + cluster linkage	B-T-X core + Beijing-adjacent + Central-southern Hebei mfg.	Cross-regional, cross-hierarchy cooperation normalizes

In 2018, cooperation intensity and scope significantly improved, forming a “Beijing-Tianjin axis + Beijing-Baoding-Shijiazhuang secondary axis” dual-axis structure. The cooperation radius expanded, and spatial attenuation effects weakened. Cities near Beijing and Tianjin, leveraging their locational advantages, became cooperation hotspots. Technology fields expanded toward biotechnology, and a trend of cooperation diversification began to emerge (7, 26). The cooperation radius extended to 200km. The “1-h traffic circle” of Beijing, Tianjin, and Baoding drove the spatial attenuation coefficient down to 0.52, meaning higher efficiency in cooperation information transmission (41). Regional industrial division of labor gradually became clearer, and innovation cooperation extended from single technology exchanges to multi-domain synergy (5, 22).

By 2023, the region-wide cooperation network was formally established. High-strength cooperation links increased significantly, with new combinations like Beijing-Xiong'an and Tianjin-Xiong'an becoming core links. Cross-regional cooperation within Hebei developed comprehensively, forming three major spatial plates: the Beijing-Tianjin-Xiong'an core cluster, the Beijing-adjacent collaboration cluster, and the Central-Southern Hebei manufacturing cluster (5, 6). Technology fields exhibited a pattern of “high-end leadership, multi-point breakthroughs,” with cooperation in gene therapy (C12N15), biological vaccines (A61K39), and medical devices (A61M) accounting for over 50%, indicating deepened cooperation in high-end biomedical fields (7, 20). This evolutionary outcome signifies that regional cooperation broke through the limitations of geographical and administrative boundaries. The pattern of industrial gradient division of labor matured, enabling efficient allocation of innovation resources across the entire region, laying a solid foundation for enhancing regional industrial competitiveness (5, 6).

### 4.4 Evolution of cooperation types and interaction modes: from single output to diversified full-chain synergy

The evolution of cooperation types adapts to the industrial division of labor characterized by “R&D in Beijing, conversion in Tianjin-Hebei,” transitioning from single R&D cooperation to a diversified coexistence of “R&D cooperation, technology transfer, and supporting services.” Interaction modes upgraded from unidirectional output to bidirectional synergy, reflecting the deepening of the regional industrial gradient division of labor and the improvement of synergy efficiency (18, 26).

In 2013, cooperation exhibited “dual-core internal closed-loop” characteristics, primarily single R&D cooperation, with only the Beijing-Tianjin pair. Technology transfer and supporting service cooperation were absent. The interaction mode manifested as unidirectional collaboration characterized by “Beijing R&D, Tianjin support” (9, 33). This pattern stemmed from the fragmentation of the regional innovation system, lacking effective cooperation carriers between core and peripheral cities, and the failure of deep connection between the innovation and industrial chains (34, 39) (Table 10).

**Table 10:** Evolutionary characteristics and key parameters of cooperation types.

Evolution stage	Year	Dominant cooperation type	Mean tech similarity	Proportion of geographic proximity (%)	Core interaction mode	Typical cooperation combinations
Single cooperation	2013	R&D Cooperation	0.5385	30.8	Unidirectional Collaboration (Beijing R&D + Tianjin Support)	Beijing-Tianjin
Diversification embryonic	2018	R&D + Technology Transfer	0.3846	46.2	Bidirectional Collaboration + Unidirectional Output	Beijing-Baoding, Tianjin-Cangzhou
Full-chain synergy	2023	R&D + Transfer + Support Services + Industrialization	0.4231	57.7	Multi-dimensional Synergy, Full-Chain Linkage	B-T-X R&D Cluster, Central-Southern Hebei Mfg. Cluster

In 2018, cooperation types began to diversify. The proportion of R&D cooperation decreased, with newly added combinations like Beijing-Baoding and Tianjin-Shijiazhuang (5 pairs). Beijing outputted pharmaceutical production technology to 6 cities including Cangzhou and Xingtai, forming a preliminary “R&D-conversion” link. Technology transfer and supporting service cooperation gradually emerged. Interaction modes shifted toward “bidirectional collaboration + unidirectional output” (17, 37). This transformation aligned with the requirements for regional coordinated development and industrial division of labor within urban agglomerations as outlined in national plans, promoting the clustering and differentiated development of strategic emerging industries like biomedicine. The technology output capacity of core cities and the undertaking capacity of peripheral cities improved simultaneously, gradually clarifying the pattern of regional industrial division of labor (2, 16).

By 2023, a diversified collaborative model has been fully established, with research and development cooperation, technology transfer, supporting services, and joint industrialization developing simultaneously. The “Beijing-Tianjin-Xiong'an” core R&D cluster formed. The BTH Technology Transfer Center precisely matched supply and demand. The BTH·Cangzhou Biomedical Industrial Park realized cross-regional cooperation with “production in Hebei, regulation belonging to Beijing.” Cross-regional industrial cooperation achieved deep integration from the technical level to the full industrial chain level (51, 52). Interaction modes upgraded to “multi-dimensional synergy, full-chain linkage” (7, 26).

The continuous deepening of typical patterns confirms the ongoing improvement of regional cooperation mechanisms. The efficiency of innovation factor flow significantly increased, regional R&D costs decreased, and conversion efficiency greatly improved (2, 37), providing strong support for high-quality industrial development. This evolutionary process showcases the “R&D-conversion-industrialization” synergy model, reflecting the inevitable trend of regional industrial gradient division of labor (14, 18).

### 4.5 Evolutionary mechanisms and summary of patterns

From 2013 to 2023, the BTH biomedical technology cooperation network underwent a three-stage evolution of “dense connection - differentiation adjustment - precise synergy,” with its core driving mechanisms exhibiting multi-dimensional dynamic characteristics (17, 18).

At the node development level, a transformation from “gradient following” to “differentiated rise” was achieved. Hebei cities transitioned from passively undertaking technology to actively participating in R&D. Some cities achieved a leap from the periphery to characteristic cores. This process is closely related to the Matthew effect in regional innovation capacity; cities with stronger innovation capacity consistently occupied the network's core positions, while non-core cities achieved innovation breakthroughs through policy support and resource accumulation (22, 23).

From the perspective of network structure evolution patterns, a shift from “unipolar radiation” to “multipolar synergy” was completed. The core pattern evolved from a single Beijing hub to a Beijing-Tianjin-Xiong'an tripod support (2, 15). The network development goal transitioned from “pursuing full coverage” to “precision and efficiency (49, 50).” The network closure effect and agglomeration effect continuously strengthened, and the circulation efficiency of innovation factors kept improving, reflecting the optimization logic of innovation networks from an industrial cluster perspective (14, 36).

From the perspective of driving mechanisms, the evolutionary dynamics exhibit multi-dimensional dynamic characteristics: geographical proximity played a key role in the initial stage, but its influence gradually weakened with the development of transportation and communication technologies. The role of social proximity and institutional proximity continuously strengthened, with trust relationships becoming the core bond for cooperation in later stages (19, 21). Policy synergy, as an important institutional guarantee, promoted the formation of a differentiated division of labor pattern - “Beijing R&D + Tianjin conversion + Hebei manufacturing” - forming a positive interaction with the regional industrial gradient (5, 6). The coupling match between patent similarity and technological strength determines the depth of cooperation: high similarity lays the foundation for cooperation, while low similarity fosters differentiated division of labor, jointly driving the network's evolution toward precise synergy (7, 34) (Table 11).

**Table 11:** Intensity of driving factors in evolution.

Driving factor	Intensity in 2013	Intensity in 2018	Intensity in 2023	Characteristics of effect
Geographical proximity	Strong (Core Driver)	Relatively Strong (Important Driver)	Medium (Auxiliary Driver)	Effect weakens with transportation improvement
Technological similarity	Medium (Local Driver)	Relatively Strong (Regional Driver)	Strong (Differentiation Driver)	High similarity promotes synergy, low similarity promotes division of labor
Policy synergy	Weak (Potential Driver)	Relatively Strong (Key Driver)	Strong (Core Driver)	Promotes optimal allocation of innovation resources
Social proximity	Weak (No significant effect)	Medium (Auxiliary Driver)	Strong (Trust Driver)	Becomes core bond for cooperation in later stages

In summary, the evolution of the BTH biomedical technology cooperation network is the result of the combined effects of node growth, structural optimization, division of labor deepening, and policy guidance, with significant improvements in regional synergy depth and specialization level (5, 17). In the future, it is necessary to further strengthen the differentiated division of labor pattern of “Beijing R&D + Tianjin conversion + Hebei manufacturing,” explore the potential for intra-Hebei cooperation, guard against lock-in effects dominated by trust relationships, expand the scope of cooperation by eliminating institutional barriers, and drive the network toward a higher quality stage of collaborative innovation (18, 38).

## 5 Spatiotemporal evolution characteristics of innovation input and output in the BTH biomedical industry

### 5.1 Spatiotemporal differentiation characteristics of input and output

#### 5.1.1 Descriptive statistical analysis results

This study employs panel data from up to 33 samples across Beijing, Tianjin, and Hebei Province to conduct a descriptive analysis of an equilibrium panel dataset spanning three regions/provinces over an 11-year observation period. The sample covers two municipalities and one province within the Jing-Jin-Ji region: Beijing, Tianjin, and Hebei Province, covering the period from 2013 to 2023 (3 municipalities/provinces × 11 years = 33 observations). The study identified three key years: 2013, 2018, and 2023, corresponding respectively to the ‘policy initiation', ‘deepening cooperation' and ‘high-quality development transformation' stages in the BTH coordinated development strategy, a conclusion consistent with the results of the network evolution analysis. Thus, *N* = 33 represents the total number of data points for all indicators in this descriptive analysis rather than the specific number of enterprises. Due to missing data for some indicators in certain years, moving average methods were employed for interpolation, resulting in varying effective sample sizes across indicators. This study focuses on two dimensions: industrial innovation inputs (R&D expenditure, full-time R&D personnel, fiscal expenditure on technology, and technology market transaction volume) and industrial innovation outputs (number of patent applications, revenue from pharmaceutical manufacturing). Utilizing five aggregate statistical indicators (minimum value, first quartile, median, third quartile, maximum value) and distribution characteristics (skewness, kurtosis), it systematically reveals structural disparities in regional industrial development. The distribution patterns of core indicators are detailed in Table 12, clearly demonstrating significant regional variations.

**Table 12:** Descriptive statistical analysis table.

Indicator	*N*	Minimum	Maximum	Mean	Std. deviation	Skewness (SK)	Kurtosis (K)
Operating revenue of pharmaceutical Mfg. (10 thousand yuan)	23.00	6,330,207.00	36,967,534.00	11,232,439.49	6,254,977.46	1.12	2.89
Total assets of pharmaceutical Mfg. (10 thousand yuan)	33.00	6,760,538.30	39,390,223.00	14,505,786.10	8,327,315.83	0.98	2.76
R&D expenditure (10 thousand yuan)	33.00	104,609.90	838,315.10	250,735.67	163,762.78	1.23	3.12
R&D personnel full-time equivalent (person-year)	33.00	2,687.00	8,127.00	4,950.21	1,120.73	0.38	2.45
Science & technology fiscal expenditure (10 thousand yuan)	33.00	455,000.00	5,219,080.00	1,892,396.18	1,487,231.94	(0.12)	2.91
Number of patent applications (pieces)	27.00	369.00	1,824.00	789.41	404.07	0.87	2.68
Technology market turnover (100 million yuan)	33.00	42.80	412.60	189.24	148.72	1.56	3.87

The sample size (*N*) varies depending on the indicator, specifically based on data availability in the statistical yearbook. The maximum sample size (*N* = 33) corresponds to a panel dataset covering three municipalities/provinces over an 11-year period (2013–2023); smaller sizes (e.g., 23 or 27) arise from missing values for certain indicators in annual observations from some cities/provinces, and even after supplementation using the moving average method, the effective sample size remains below the full sample size of 33.

#### 5.1.2 Input indicators: coexistence of scale differentiation and allocation imbalance

The R&D expenditure of leading cities (e.g., Beijing) is approximately 8.01 times that of the lagging ones, indicating a high concentration of financial resources. The full-time equivalent number of R&D personnel follows a nearly symmetrical distribution (skewness 0.38), yet the range of extreme values spans as high as 5,440 person-years. The median value for the Beijing sample exceeds that of Hebei Province by 26.4%, reflecting the distinct characteristic of talent concentration in northern regions. Science and technology fiscal expenditure approaches a normal distribution (skewness = −0.12) with minimal regional disparities, suggesting that policy efforts focus on foundational support. Technology market transaction volumes demonstrate a clear right-skewed distribution (skewness 1.56): Beijing accounts for 62.3% of total technology transactions across all three regions, establishing a unidirectional technology flow pattern with Tianjin and Hebei.

#### 5.1.3 Output indicators: worsening differentiation between innovation achievements and economic benefits

The number of patent applications shows a right-skewed distribution (skewness 0.87), with a gap of 4.9 times between maximum and minimum values. The discrete coefficient is higher than the mean of input indicators, indicating that input differentiation has transformed into significant divergence in innovation output. The skewness of operating revenue of pharmaceutical manufacturing is 1.12. The median for Beijing samples is 39.1% and 83.9% higher than that of Tianjin and Hebei, respectively, directly mapping the regional economic strength gap onto the level of industrial development.

Over time, R&D expenditure shows a linear growth trend (Y = 182.3 + 19.7t, R^2^ = 0.92), with a growth rate jumping to 12.5% in 2020, highly coinciding with policy nodes of coordinated development. The number of patent applications increased by 31.1% after policy implementation in 2020, with Hebei's growth rate (15.2%) higher than Beijing's (9.8%), indicating a gradual narrowing of the regional innovation gap. The growth of operating revenue lags behind patent numbers by about 2 years, conforming to the 3–5 year cycle law of “R&D-clinical-commercialization.” After 2022, the growth rate rose to 10.8%, with Hebei's revenue growth rate (13.5%) surpassing Beijing's, demonstrating the benefits of industrial gradient transfer.

### 5.2 Core constraining factors

The core constraining factors for the high-quality development of the BTH biomedical industry exhibit significant hierarchical characteristics (Table 13): low R&D personnel efficiency (obstacle degree 18.7%), insufficient R&D expenditure (15.3%), and low patent revenue conversion rate (12.1%) are the three primary obstacles. While sharing commonalities with the Yangtze River Delta region, these factors also highlight the specific problems of resource allocation imbalance and insufficient conversion efficiency in the BTH region.

**Table 13:** Obstacle degree analysis table.

Primary indicator	Obstacle degree (G)	Core obstacle secondary indicator	Obstacle degree (G)
Industrial efficiency B	32.60%	R&D Personnel Efficiency (b2)	18.70%
Industrial innovation D	28.90%	Insufficient R&D Expenditure (d1)	15.30%
Industrial benefit C	24.50%	Patent Revenue Conversion Rate (c2)	12.10%
Industrial scale A	14.00%	Insufficient Enterprise Agglomeration (a1)	7.80%

List only the top four secondary indicators by obstacle severity ranking.

### 5.3 Driving mechanisms of network evolution

The evolution of the BTH biomedical technology cooperation network is a complex process driven by multiple factors. Changes in its morphology and structure are influenced by the combined effect of three factors: exogenous attributes, endogenous structure, and network basic characteristics. Exogenous attributes mainly refer to external conditions such as regional policy environment, differences in resource endowment, and market openness. Endogenous structure focuses on internal characteristics among network nodes, such as connection strength, reciprocity, and distribution of structural holes. Network basic characteristics include overall topological properties like network size, density, and centrality. These three types of factors intertwine and dynamically influence each other, causing network evolution to exhibit significant dynamic adaptability: the network continuously adjusts its connection patterns and spatial structure based on changes in internal and external conditions to achieve optimization of resource allocation and innovation efficiency.

To systematically reveal the specific influence of the aforementioned factors on network evolution, this study employs the TERGM regression model for empirical testing. Following the academic convention of presenting core results in tabular form (17), the regression analysis results are clearly summarized. Table 14 includes the regression coefficients for each explanatory variable, the Bootstrap sampling mean, and its 95% confidence interval, aiming to demonstrate the robustness of the model results and statistical significance while presenting the estimates, thus providing a reliable basis for subsequent discussion. The specific results are summarized in the following table.

**Table 14:** TERGM regression results.

Variable name	Coefficient (Estimate)	Bootstrap mean (Bootmean)	95% CI (2.50%)	95% CI (97.50%)	Significance judgment
Edge density (edges)	−2.301	−2.538	−5.987	−1.981	^***^
Reciprocity (mutual)	0.849	0.103	−19.601	1.955	Marginally significant
Economic endowment (gdp)	0.002	0.003	0.001	0.008	^**^
Policy support (gov_exp)	−0.003	−0.003	−0.009	0.01	Not significant

^***^ *p* < 0.001, ^**^ *p* < 0.01, ^*^ *p* < 0.05; Marginally significant means that although the confidence interval crosses zero, the coefficient direction is consistent with theoretical expectations, and the Bootstrap mean is stable. Model fit and convergence diagnostics (see Table 3) confirmed that the model converged successfully (Geweke diagnostic < |1.96| for all parameters) and that the observed network statistics fell within the 95% confidence intervals of the simulated distributions, indicating no degeneracy and acceptable goodness-of-fit.

#### 5.3.1 Exogenous attribute drivers: economic endowment as the core driver, policy support effect needs optimization

Based on empirical model analysis, this study identifies the level of economic development as the core driver for the expansion of the BTH biomedical technology cooperation network. Regression results show that the coefficient for city GDP is significantly positive (0.002, *p* < 0.01). This indicates that, controlling for other variables, for every 10,000 yuan increase in a city's economic development level, the average probability of its participation in forming cross-regional technology cooperation relationships increases by a factor of 0.002. This finding statistically validates the supporting role of the economic base for innovation linkages: cities with stronger economic strength, like Beijing and Tianjin, leverage their deep capital accumulation, well-developed research infrastructure, and active market entities, are better equipped to undertake high-risk, high-investment joint R&D projects, thus consistently playing core hub roles in the network. This result also empirically confirms that technology cooperation is essentially a resource integration behavior supported by economic strength, indicating that regional collaborative innovation relies not only on knowledge complementarity but also on sustained investment of economic resources and market momentum. In contrast, the policy variable of science and technology fiscal expenditure did not exhibit the expected promoting effect. Its regression coefficient is negative (−0.003) and statistically insignificant, suggesting that current fiscal technology investments have not significantly stimulated inter-regional cooperation willingness. The core reason for this phenomenon lies in the obvious tendency toward “ each acting on its own” in the science and technology policies of the BTH three regions: Beijing's fiscal input focuses on supporting cutting-edge basic research and original innovation, Tianjin's resources are more directed toward achievement conversion and platform construction, while Hebei leans toward industrial undertaking and implementation subsidies. This lack of cross-regional coordination in the fiscal support system means government funds fail to form a synergistic force functionally. Instead, it may exacerbate resource barriers between regions due to misaligned policy goals and inconsistent support standards, potentially even inducing local protectionist tendencies locally, thereby weakening rather than promoting overall regional cooperation willingness. This suggests that without systematic integration and guidance of science and technology fiscal resources from a regional synergy perspective, simply increasing funding may not translate into momentum for network linkages and could even be counterproductive due to institutional fragmentation.

#### 5.3.2 Endogenous structure evolution: reciprocity as a key bond for network synergy

Regression analysis further reveals that internal structural characteristics of the network also profoundly influence cooperation dynamics. The coefficient for reciprocity is positive (0.849) and marginally statistically significant, with its Bootstrap mean stable at 0.103. This confirms that reciprocal relationships are a key endogenous structural force driving network evolution. This result indicates that establishing bidirectional and stable collaborative relationships between nodes significantly increases the likelihood of future cooperation, thus serving as an important mechanism for the solidification and expansion of the network structure.

The strengthening of this reciprocity mechanism is intuitively reflected in the diachronic transformation of technology cooperation models in the BTH region. Specifically, the cooperation mode has gradually shifted from “unidirectional technology output” from Beijing to Tianjin, Shijiazhuang, etc., in 2013, to the emergence of “bidirectional collaborative R&D” between multiple city pairs such as Tianjin-Cangzhou and Shijiazhuang-Baoding post-2018. This transformation means that participants establish stable trust and normative mechanisms through repeated interactions, forming a positive feedback loop of “cooperation - gaining benefits - strengthening trust - cooperating again.”

From the perspective of the overall network morphology, the generalization of reciprocal relationships is effectively driving the regional innovation structure to evolve from an early “core monopoly” pattern dominated by a single or a few cores toward a “multi-point linkage” network where multiple nodes interconnect and collaborate. This is not merely an increase in the number of connections but an enhancement in the quality and stability of cooperation, laying a structural foundation for the resilience and sustainability of the regional innovation ecosystem.

#### 5.3.3 Network basic characteristics: negative coefficient of edge density reflects cooperation threshold

Regression results show that the coefficient for edge density is significantly negative (−2.301, *p* < 0.001). This statistical characteristic highly aligns with the prevalent “sparsity” of technology cooperation networks in biomedicine, reflecting the naturally high threshold for cross-entity cooperation in this field. This primarily stems from the inherent characteristics of biomedical R&D itself: long cycles, high investment, and strong specificity. The entire process from basic research, preclinical development to clinical trials and approval for market launch is typically time-consuming with uncertain outcomes. Simultaneously, issues concerning intellectual property ownership and the distribution of benefits from achievement conversion are relatively complex, making potential partners cautious when establishing links, resulting in a generally low overall network connection density.

However, through diachronic analysis of the regression results, it was found that the absolute value of the negative coefficient for edge density showed a gradual decreasing trend between 2013 and 2018. This change indicates that cooperation barriers within the BTH region are systematically lowering, and network connectivity is continuously improving. The driving force primarily stems from two aspects: first, the substantive advancement of transportation integration, significantly shortening the constraints of geographical distance on the exchange of personnel, samples, and knowledge; second, the continuous and detailed implementation of regional coordinated development policies, creating a more convenient institutional environment in areas such as platform co-construction, data sharing, and regulatory mutual recognition. The combined effect of these factors effectively promotes the cross-regional flow of information and resources, making it easier for potential cooperation to materialize into actual linkages, thereby driving the network structure to evolve from relatively loose toward a more closely-knit and stable direction.

## 6 Discussion

### 6.1 Public health implications of network evolution

The evolution of the BTH biomedical technology cooperation network from a “dual-core monopoly” to a “Beijing-Tianjin-Xiong'an triangular structure” has significantly improved network density and reciprocity. According to policy network studies, such structural improvements are positively associated with system performance (13). In public health terms, this means that knowledge spillovers from Beijing's basic research to Hebei's manufacturing are becoming faster and more reliable, which directly benefits the translation of innovations into clinical applications. Moreover, a resilient innovation network underpins the synergistic security of regional medical resources, as demonstrated by comparative studies of the BTH, YRD, and PRD city networks (4).

However, the persistent obstacles identified in this study: low efficiency of R&D personnel (18.7%) and a low conversion rate of patent income (12.1%), directly indicate that many biomedical innovations remain in the preclinical stage for far longer than necessary (48). As Lurie et al. (10) demonstrated during the COVID-19 pandemic, every month saved in the R&D-to-approval process can have a measurable impact on mortality. Therefore, we propose policy interventions to bridge the gap between research and development, translation, and industrialization, which falls not only within the scope of industrial policy but is also a crucial public health strategy.

The public health significance of our findings extends far beyond industrial competitiveness. The identified barriers (low research efficiency, insufficient patent commercialization, and chain disconnections) fundamentally obstruct the timely delivery of diagnostics, vaccines, and therapeutics to patients. As Wang et al. (3) noted, efficient biotechnology collaboration networks are crucial for rapid pathogen identification, early disease detection, and vaccine development: capabilities essential for responding to public health emergencies. Conversely, fragmented networks delay knowledge dissemination and hinder the translation of research outcomes into medical interventions. Cai et al. (11) showed in the food industry that network fragmentation (density as low as 0.0005) severely limits knowledge spillovers. Extending this to biomedicine, the persistent “core-periphery” structure in the BTH region implies that peripheral cities (e.g., several prefecture-level cities in Hebei) may experience systemic delays in accessing cutting-edge innovations from Beijing and Tianjin, potentially leading to uneven health outcomes across the region.

### 6.2 From network optimization to health equity: a causal pathway

Based on our empirical findings and the theoretical framework outlined in Figure 1, we propose an empirically testable causal pathway linking innovation network optimization to public health equity:

Increased innovation network density → Elevated cross-regional patent conversion rate → Reduced drug production costs → Decreased patient out-of-pocket expenditures → Improved regional health equity

This pathway aligns with established health economics literature on vaccine pricing and incentive mechanisms (30) as well as hierarchical medical system policies (32). Specifically, Villota-Miranda and Rodríguez-Ibeas (30) demonstrate that efficient innovation-to-market processes directly lower vaccine costs and improve accessibility. Similarly, Hu et al. (32) show that coordinated service delivery reforms can reduce interregional disparities in health outcomes. Translating these insights to the BTH biomedical context, we argue that policy interventions designed to bridge the “R&D-conversion-industrialization” gaps should be evaluated not only by economic returns but also by health impact metrics such as time-to-approval, interregional price variation, and disease-specific mortality. Preliminary evidence from cross-regional comparisons already suggests that cities with denser innovation and policy networks exhibit higher medical resource synergy resilience (4).

Future research should directly quantify these links using drug price databases, insurance claims, or mortality data for targeted diseases (e.g., certain cancers or infectious diseases). Such empirical validation would strengthen the evidence base for viewing innovation network policies as public health interventions.

### 6.3 Integrated analysis of network and obstacle models

A key contribution of this study is the explicit linkage between identified obstacles and the network's structural limitations. The obstacle degree model identifies “low efficiency of R&D personnel” as the single largest barrier (18.7%). This finding directly explains the observed sparsity and core-periphery structure in the TERGM analysis: peripheral cities like those in Hebei lack the high-quality R&D talent needed to form substantive collaborative ties with core nodes such as Beijing.

Conversely, the TERGM result that policy support (gov_exp) is not statistically significant (and negatively signed) helps explain why the obstacle of “insufficient patent revenue conversion” (12.1%) persists. Current fiscal policies focus on R&D inputs rather than outputs or cross-regional coordination, failing to stimulate the reciprocal (mutual) relationships that TERGM identifies as crucial for network expansion and stability. This integrated perspective reveals that policy interventions should shift from simply increasing R&D funding toward specifically targeting the quality of R&D personnel in peripheral regions and the efficiency of cross-regional technology transfer platforms.

In summary, the obstacle degree model identifies what constrains the system (e.g., personnel inefficiency, weak patent conversion), while TERGM explains why these constraints manifest as a sparse, core-periphery network structure (low reciprocity, non-significant policy effects). The causal chain is as follows: a shortage of micro-level R&D talent at the edge nodes reduces the likelihood of forming bidirectional connections, which in turn sustains the hierarchical network structure and limits cross-regional spillovers, ultimately leading to low macro-level efficiency in converting patents into economic and public health returns. This integrated logic provides a unified theoretical basis for the policy recommendations that follow.

### 6.4 Theoretical contributions and limitations

This study makes two main theoretical contributions. First, it constructs a coupled analytical framework of “innovation input → network evolution → output efficiency,” addressing the deficiency of single-dimension analyses in existing regional biomedical industry research. Second, it reveals the differentiated rise path of non-core cities under the “core-periphery” structure, enriching the theoretical understanding of regional innovation network evolution in the context of public health translation.

Several limitations should be acknowledged. The sample selection focused on key enterprises and core cities, with insufficient coverage of small- and medium-sized enterprises and county-level actors. The analysis of micro-level behavioral mechanisms of network actors (e.g., firms, research institutions) needs further deepening. Moreover, although we took methodological precautions to ensure the robustness of TERGM estimates (bootstrapping, convergence diagnostics, goodness-of-fit assessment), the relatively small number of network nodes (13) remains a potential limitation. Future research should expand the sample scope to include more granular intra-city actors (e.g., industrial parks, research institutions) and employ qualitative methods such as enterprise interviews to analyze how firm heterogeneity and digital technologies affect network evolution and the integration of innovation and industrial chains (53). Additionally, future studies should directly measure public health outcomes (e.g., drug approval times, interregional price differences, vaccine coverage rates) to validate the causal pathway proposed in Section 6.2.

## 7 Conclusions and recommendations

Based on the research conclusions, to promote the high-quality coordinated development of the BTH biomedical industry and in line with the gradient division of labor pattern of “Beijing R&D, Tianjin conversion, Hebei manufacturing,” the following systematic recommendations are proposed:

### 7.1 Strengthen regional synergy mechanisms and build a precise division of labor system

Leveraging the support of the “Beijing-Tianjin-Hebei” triangular core, establish a cross-regional collaborative governance platform to clarify the functional positioning and division of labor boundaries of the three regions: Beijing focuses on original innovation and core technology breakthroughs, enhancing the radiative and leading role of national key laboratories; Tianjin deepens its R&D conversion hub function, improving the construction of carriers such as pilot-scale test bases and clinical trial platforms; Hebei focuses on enhancing advanced manufacturing capacity, undertaking industrialization projects and supporting services. Establish a benefit-sharing mechanism for patent conversion and a cross-regional cooperation assessment and evaluation system to break down administrative barriers restricting factor flow and promote the efficient optimal allocation of innovation resources across the entire region. From a public health perspective, this precise division of labor accelerates the pipeline from basic research to clinical application, ensuring that new diagnostics and therapeutics reach patients more quickly and equitably across the BTH region.

### 7.2 Improve the innovation ecosystem chain and bridge the “R&D-conversion-industrialization” gaps

Targeting the shortcomings in industrial efficiency, focus on building a full-chain innovation service system: strengthen the construction of technology trading platforms and intermediary organizations, promote the precise transfer of Beijing-Tianjin technological achievements to Hebei, fill the gaps in Hebei's pilot-scale testing links and approval resources, support cross-regional co-construction of clinical trial institutions and industrialization bases, and cultivate specialized supporting service formats such as intellectual property services and business incubation to reduce the cost of transforming innovation achievements. Enhance policy coordination from the “R&D end” to the “conversion end” and the “industrialization end,” extending fiscal support from single R&D funding to conversion links and supporting services, thereby improving the mediating transmission effect of the technology market. By bridging these gaps, the proposed measures reduce the time lag between patent approval and market availability of essential medicines, directly benefiting population health and strengthening the region's capacity to respond to health emergencies.

### 7.3 Optimize factor allocation efficiency and resolve structural mismatches

Addressing the contradiction of imbalanced talent structure and uneven funding allocation, implement differentiated factor empowerment strategies: Beijing focuses on attracting high-end R&D talent to alleviate the “ample funds but shortage of high-end talent” situation; Hebei optimizes its R&D personnel structure through skill training and cross-regional talent exchanges, reducing the proportion of experimental technicians and increasing the proportion of R&D engineers. Establish a BTH R&D resource sharing platform to promote the cross-regional sharing of research instruments, data resources, and talent teams, achieving a coordinated match of “funds, talent, and industry” and enhancing the overall innovation input efficiency of the region. Efficient allocation of resources ensures that research talent and financial resources are directed toward areas capable of generating the greatest health benefits, whether in basic research in Beijing, technology transfer platforms in Tianjin, or manufacturing capacity in Hebei, rather than becoming entangled in mismatched regional resource pools.

### 7.4 Implement precise policy support and strengthen innovation network resilience

Based on the characteristics of the network evolution stage and regional gradient differences, formulate differentiated policy toolkits: For core nodes like Beijing-Tianjin-Xiong'an, focus on supporting cooperation in high-end innovation fields and generic technology R&D; for potential nodes in Hebei, increase investment in infrastructure and carrier construction to enhance their capacity to receive radiation (54). Improve policy coordination mechanisms, unify regional industrial standards, market access, and regulatory rules, and facilitate the normalization of cross-regional cooperation models such as “production in Hebei, regulation belonging to Beijing.” Simultaneously, strengthen dynamic policy adjustment and evaluation feedback to prevent network lock-in effects under the dominance of trust relationships, driving the innovation network toward a more open and efficient collaborative stage (46). Ultimately, these differentiated policies aim to build a resilient innovation ecosystem that can sustain rapid health technology development even under external shocks, such as pandemics or supply chain disruptions, thereby safeguarding long-term public health security.

From a health systems perspective, the proposed division of labor (“Beijing for R&D, Tianjin for conversion, Hebei for manufacturing”) is expected to shorten the average R&D-to-registration cycle for novel therapies by reducing redundant pilot-scale testing and streamlining cross-regional regulatory approval pathways. Faster approval times, coupled with Hebei's manufacturing scale, can lower production costs and increase the affordability of essential medicines for the region's 110 million residents. Future research should quantify these health benefits directly. For example, by tracking changes in drug pricing, insurance coverage rates, or mortality from targeted diseases (e.g., certain cancers or infectious diseases) following network optimization.

## Data Availability

The data that support the findings of this study are available from the corresponding author upon reasonable request. Patent data were sourced from the incoPat global patent database (https://www.incopat.com/), a commercial database. Enterprise panel data and city-level data were compiled from the Beijing, Tianjin, and Hebei Statistical Yearbooks (2013–2023). The patent data are not publicly available due to commercial restrictions; the statistical yearbook data are publicly accessible but were compiled by the authors from the original sources. Requests to access the datasets should be directed to the corresponding author at tanqingli@gdpu.edu.cn.
